# Neuroinflammation increases GABAergic tone and impairs cognitive and motor function in hyperammonemia by increasing GAT-3 membrane expression. Reversal by sulforaphane by promoting M2 polarization of microglia

**DOI:** 10.1186/s12974-016-0549-z

**Published:** 2016-04-18

**Authors:** Vicente Hernandez-Rabaza, Andrea Cabrera-Pastor, Lucas Taoro-Gonzalez, Alba Gonzalez-Usano, Ana Agusti, Tiziano Balzano, Marta Llansola, Vicente Felipo

**Affiliations:** Laboratorio de Neurobiología, Centro Investigación Príncipe Felipe , Eduardo Primo Yúfera, 3, Valencia, 46012 Spain

**Keywords:** Hyperammonemia, Hepatic encephalopathy, Neuroinflammation, Microglia, Sulforaphane, GAT-3, GABA, Motor in-coordination, Learning

## Abstract

**Background:**

Hyperammonemia induces neuroinflammation and increases GABAergic tone in the cerebellum which contributes to cognitive and motor impairment in hepatic encephalopathy (HE). The link between neuroinflammation and GABAergic tone remains unknown. New treatments reducing neuroinflammation and GABAergic tone could improve neurological impairment. The aims were, in hyperammonemic rats, to assess whether:

- Enhancing endogenous anti-inflammatory mechanisms by sulforaphane treatment reduces neuroinflammation and restores learning and motor coordination.
- Reduction of neuroinflammation by sulforaphane normalizes extracellular GABA and glutamate-NO-cGMP pathway and identify underlying mechanisms.
- Identify steps by which hyperammonemia-induced microglial activation impairs cognitive and motor function and how sulforaphane restores them.

**Methods:**

We analyzed in control and hyperammonemic rats, treated or not with sulforaphane, (a) learning in the Y maze; (b) motor coordination in the beam walking; (c) glutamate-NO-cGMP pathway and extracellular GABA by microdialysis; (d) microglial activation, by analyzing by immunohistochemistry or Western blot markers of pro-inflammatory (M1) (IL-1b, Iba-1) and anti-inflammatory (M2) microglia (Iba1, IL-4, IL-10, Arg1, YM-1); and (e) membrane expression of the GABA transporter GAT-3.

**Results:**

Hyperammonemia induces activation of astrocytes and microglia in the cerebellum as assessed by immunohistochemistry. Hyperammonemia-induced neuroinflammation is associated with increased membrane expression of the GABA transporter GAT-3, mainly in activated astrocytes. This is also associated with increased extracellular GABA in the cerebellum and with motor in-coordination and impaired learning ability in the Y maze. Sulforaphane promotes polarization of microglia from the M1 to the M2 phenotype, reducing IL-1b and increasing IL-4, IL-10, Arg1, and YM-1 in the cerebellum. This is associated with astrocytes deactivation and normalization of GAT-3 membrane expression, extracellular GABA, glutamate-nitric oxide-cGMP pathway, and learning and motor coordination.

**Conclusions:**

Neuroinflammation increases GABAergic tone in the cerebellum by increasing GAT-3 membrane expression. This impairs motor coordination and learning in the Y maze. Sulforaphane could be a new therapeutic approach to improve cognitive and motor function in hyperammonemia, hepatic encephalopathy, and other pathologies associated with neuroinflammation by promoting microglia differentiation from M1 to M2.

## Background

Hepatic encephalopathy (HE), both clinical and minimal (MHE), is an important problem which affects several million patients with liver cirrhosis worldwide [1]. Patients with MHE show psychomotor slowing, attention deficits, mild cognitive impairment, and impaired visuo-motor coordination which reduce the quality of life and the ability to perform daily life tasks [2, 3].

MHE and HE also pose a relevant economic burden by increasing the risk of traffic-, work-, and home accidents, and the number of falls and hospitalizations. Early treatment of patients with HE would improve their quality of life and life span and reduce accidents, hospitalizations, and associated costs.

Current treatments for HE are mainly directed to reduce ammonia levels by using non-absorbable disaccharides or antibiotics such as rifaximin, which also reduces inflammation. These treatments are not completely effective likely because they do not eliminate hyperammonemia or inflammation which is continuously generated by the liver disease. Therefore, it is necessary to look for new treatments acting on a different target involved in the mechanisms by which liver failure leads to MHE [4].

Hyperammonemia and inflammation play a synergistic role in the induction of the cognitive and functional alterations in patients with liver cirrhosis and MHE [5–7]. Chronic moderate hyperammonemia, similar to that present in cirrhotic patients, is enough to induce neuroinflammation and neurological alterations in rats without liver failure [4, 8]. Although liver failure is the main cause of hyperammonemia, it also occurs in many other pathological situations, including congenital defects in enzymes of the urea cycle [9], in children with perinatal asphyxia or preterm delivery [10, 11], Reye’s syndrome [12], hyperinsulinism-hyperammonemia syndrome [13], organic acidemias, and carnitine deficiencies [14]. Hyperammonemia may also arise as complications of valproate therapy, hemodialysis, or leukemia treatment [15, 16]. These situations are associated with hyperammonemic encephalopathy [17].

Chronic hyperammonemia induces neuroinflammation through activation of microglia [8, 18].

The microglial activation marker Iba-1 is also up-regulated in the cerebral cortex from acutely ammonia-intoxicated rats [19]. This indicates that hyperammonemia per se is enough to induce microglial activation and neuroinflammation.

Neuroinflammation mediates the cognitive and motor alterations found in rats with hyperammonemia and HE and also in patients with different neurological or neurodegenerative diseases. Treatment of hyperammonemic rats or of rats with HE due to portacaval shunts with anti-inflammatories such as ibuprofen or MAP kinase p38 inhibitors reduces neuroinflammation and restores cognitive and motor function [8–18, 20, 21]. This suggests that similar treatments would be useful in patients with HE. However, both ibuprofen (and other nonsteroidal anti-inflammatory agents, NSAIDs) and inhibitors of MAP kinase p38 have secondary effects which precludes its therapeutic use to treat the cognitive and motor alterations in HE. NSAIDs should be avoided in cirrhotic patients because of risk of renal impairment, hepatorenal syndrome, and gastrointestinal hemorrhage [22]. Concerning p38 inhibitors, several different molecules have been evaluated in phase 2 trials. Unfortunately, clinical efficacy was not observed, and dose-related toxicity was seen [23].

Chronic hyperammonemia induces neuroinflammation through activation of microglia [8, 20]. In this work, we hypothesized that enhancing the endogenous mechanisms aiming to reduce microglial activation would be useful to reduce neuroinflammation and restore neurotransmission and cognitive and motor function in hyperammonemia and HE. A good target to reach this aim seems to be the Nrf2 system, which has been proposed as a therapeutic target against brain inflammation [24]. The activity of the endogenous Nrf2 system may be enhanced by some small molecules such as sulforaphane (SFN), a natural compound derived from cruciferous vegetables such as broccoli, which dissociates Nrf2 from keap-1, promoting its translocation to the nucleus and enhancement of antioxidant and anti-inflammatory responses [24–26]. Sulforaphane reduces lesion progression, infarct volume, and neurological dysfunction in rodents with ischemic stroke [27, 28] and is also neuroprotective in experimental parkinsonism [29].

In this work, we have used a model of chronic hyperammonemia without liver failure. It has been shown that most effects induced by chronic hyperammonemia (including neuroinflammation) are also present in rats with liver failure (e.g., [8]). This model has been recommended by the International Society for Hepatic Encephalopathy to assess the contribution of hyperammonemia to HE [30].

The first aim of this study was therefore to assess whether enhancing endogenous anti-inflammatory mechanisms by chronic treatment of hyperammonemic rats with SFN reduces activation of microglia and neuroinflammation and restores learning ability.

Neuroinflammation impairs cognitive and motor function by altering neurotransmission. In HE, hyperammonemia-induced alterations in GABAergic neurotransmission contribute to the neurological alterations [4, 31–33]. Chronic hyperammonemia increases extracellular GABA in cerebellum leading to reduced function of the glutamate-nitric oxide (NO)-cGMP pathway which, in turn, impairs the ability to learn a Y maze. Blocking GABA_A_ receptors with bicuculline reduces GABAergic tone and restores the function of the pathway and the ability to learn the Y maze task [32].

As the anti-inflammatory treatments with ibuprofen or inhibitors of p38 mentioned above also restore the function of the pathway and learning ability [9, 20], this suggests that neuroinflammation may be enhancing extracellular GABA in cerebellum which would mediate the impairing effects of neuroinflammation on the glutamate-NO-cGMP pathway and learning. A second aim of this study was to assess whether reduction of neuroinflammation in hyperammonemic rats by SFN normalizes in cerebellum in vivo the glutamate-NO-cGMP pathway and extracellular GABA concentration and analyze possible underlying mechanisms.

Increased GABAergic tone also induces motor in-coordination and extracellular GABA in cerebellum correlates with motor in-coordination in rats [34]. Gonzalez-Usano et al. [33] showed that treatment with pregnenolone sulfate reduces extracellular GABA and restores motor coordination in hyperammonemic rats. Taking into account these reports, a third aim of this study was to assess whether treatment with SFN also restores motor coordination in hyperammonemic rats.

The last aim of this work was to propose a possible sequence of the main steps involved in the process by which hyperammonemia-induced microglial activation leads to cognitive impairment and motor in-coordination and how SFN restores them.

To reach the above aims, we analyzed in control and hyperammonemic rats, treated or not with SFN, (a) the ability to learn a conditional discrimination task in the Y maze; (b) motor coordination in the beam walking; (c) the function of the glutamate-NO-cGMP pathway and extracellular GABA by in vivo microdialysis in freely moving rats; (d) microglial activation, by analyzing by immunohistochemistry or Western blot different pro- and anti-inflammatory markers (IL-1b, Iba1, IL-4, IL-10, Arg1, YM-1); and (e) we also assessed whether increased extracellular GABA is associated with enhanced expression in membrane of the GABA transporter GAT-3.

## Methods

### Model of chronic hyperammonemia in rats

Male Wistar rats (120–140 g, Charles River, France) were made hyperammonemic by feeding them an ammonium-containing diet as in [35]. The experiments were approved by the Center and carried out in accordance with the European Communities Council Directive (86/609/EEC).

### Treatment with sulforaphane

Rats were treated daily with sulforaphane (1-Isothiocyanate-4-methylsulfinylbutane; LKT Laboratory, St. Paul, MN, USA) or saline. Sulforaphane (SFN) in sterile saline was injected intraperitoneally at 0.5 mg/kg per day. The dose was chosen based on previous studies in the literature [36]. Treatment started 2 weeks after the ammonium-containing diet and was maintained during all experiments. Injections were performed in sterile conditions by expert individuals. In spite of long-term i.p. injections, no complications such as infections, pain, or needle-induced were observed. Sulforaphane did not affect body weight or food consumption in control or hyperammonemic rats [37].

**Fig. 1:**
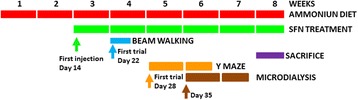
Scheme showing the experimental design

The experimental design is summarized in Fig. 1. The beam walking test was performed during 3 days beginning on day 22 of hyperammonemia. The microdialysis experiments were performed during seven working days beginning on day 35 of hyperammonemia. The sacrifice was performed during 4 days, beginning on day 49 of hyperammonemia. These manipulations were not performed exactly on the same day in all rats because it is technically impossible. In all cases, the manipulations were performed each day in the same number of rats from each group (control; control + sulforaphane, hyperammonemia, and hyperammonemia + sulforaphane). Although the severity of encephalopathy or the effects on microdialysis parameters may change with time, these changes occur slowly and remain similar for more than 2 weeks. The results obtained in the different individuals are therefore comparable.

### Learning of a conditional discrimination task in a Y maze

Learning ability was tested as in Aguilar et al. [38] in a wooden Y-shaped maze. Rats must learn where the food is depending on the color of the walls. Rats performed 10 trials per day, until the completion of a criterion of 10 correct responses in the same day or a maximum of 250 trials.

### Motor coordination: beam walking test

Motor coordination was tested using a wood strip (20 mm diameter) as described by Gonzalez-Usano et al. [33]. Rats are made to go through a 1-m-length wooden stick located approximately 1 m above the ground, and two observers count the number of slips committed by the rats. The number of foot faults (slips) is recorded as a measure of in-coordination.

### In vivo microdialysis

Rats were anesthetized using isoflurane, and a microdialysis guide was implanted in the cerebellum (AP −10.2, ML −1.6, and DV −1.2), as in [29]. After 48 h, a microdialysis probe was implanted in the freely moving rat. Probes were perfused (3 μl/min) with artificial cerebrospinal fluid: (in mM): NaCl, 145; KCl, 3.0; CaCl_2_, 2.26; buffered at pH 7.4 with 2 mM phosphate. After a 2–3 h stabilization period, samples were collected every 30 min. When indicated, NMDA (0.5 mM) was administered to activate the glutamate-NO-cGMP pathway [39]. Samples were made 4 mM in EDTA and stored at −80 °C until analysis of cGMP or GABA content.

### cGMP determination

cGMP was measured with an enzyme immunoassay kit from Amersham (Amersham Biotrak GE Healthcare) using 50 μl of dialysate from cerebellar microdialysis experiments.

### GABA determination

To assess the basal level of extracellular GABA in the cerebellum, five microdialysis samples were collected before addition of NMDA. GABA concentration was measured by HPLC as described by Canales et al. [40].

Membrane surface expression of the GAT-3 transporter by cross-linking with BS_3_ in cerebellar slices was analyzed as described by Boudreau and Wolf [41]. Control and hyperammonemic rats were decapitated and their brains transferred into ice-cold Krebs buffer (in mmol/L): NaCl 119, KCl 2.5, KH_2_PO_4_ 1, NaHCO_3_ 26.2, CaCl_2_ 2.5, and glucose 11, aerated with 95 % O_2_ and 5 % CO_2_ at pH 7.4. The cerebellum was dissected and transversal slices (400 μm) were obtained using a vibrotome. Slices were added to tubes containing ice-cold standard buffer with or without 2 mM BS_3_ (Pierce, Rockford, IL) and incubated for 30 min at 4 °C. Cross-linking was terminated by adding 100 mM glycine (10 min, 4 °C). The slices were homogenized by sonication for 20 s. Samples treated or not with BS_3_ were analyzed by Western blot using anti-GAT-3 (1:2000; Abcam, Cambridge, UK). The surface expression of GAT-3 was calculated as the difference between the intensity of the bands without BS_3_ (total protein) and with BS_3_ (non-membrane protein).

### Analysis of protein content in the cerebellum by Western blot

The animals were sacrificed by decapitation and the cerebellums were dissected and homogenized in 66 mM Tris–HCl (pH 7.4), 1 % SDS, 1 mM EGTA, 10 % glycerol, 1 mM sodium ortho-vanadate, and 1 mM sodium fluoride containing protease inhibitor cocktail (Roche, Mannheim, Germany). Samples were subjected to electrophoresis and immunoblotting as in Felipo et al. [42]. Primary antibodies were against IL-4, IL-10, iba-1, Ym-1, and GAT 3 (1:2000) from Abcam (Cambridge, UK); IL-1β (1:500) from R&D SYSTEMS (Minneapolis, USA); Arg-1 from BD Bioscience (NJ, USA); and glial fibrillary acidic protein (GFAP) (1:5000) from Sigma (St. Louis, MO, USA). As a control for protein loading, the same membranes were also incubated with anti-actin (Abcam, Cambridge, MA; 1:1000). Secondary antibodies were anti-rabbit, anti-goat, or anti-mouse IgG (1:2000) conjugated with alkaline phosphatase (Sigma, St. Louis, MO). The images were captured using the ScanJet 5300C (Hewlett- Packard, Amsterdam, the Netherlands) and band intensities quantified using the Alpha Imager 2200, version 3.1.2 (AlphaInnotech Corporation, San Francisco).

### Immunohistochemistry

Coronal 30-μm sections were cut on a cryostat and stored at 4 °C in PB with 0.1 % azide until further processing. Free-floating sections were washed, endogenous peroxidase activity was quenched with 3 % H_2_O_2_ for 15 min, and sequential incubations with blocking serum (normal goat serum or horse serum) and primary antibodies (overnight 4 °C) were performed. For double immunofluorescence, corresponding antibodies were incubated together. Primary antibodies were against IL-4, IL-10, iba-1, and GAT 3 from Abcam (Cambridge, UK); IL-1β from R&D SYSTEMS (Minneapolis, USA); and glial fibrillary acidic protein (GFAP) (1:5000) from Sigma (St. Louis, MO). Incubation with biotinylated secondary antibodies and with avidin-biotin-HRP complex (ABC kit, Vector, CA, USA) followed. The stained sections were mounted on slides, dehydrated, and coverslipped. For immunofluorescence, secondary antibodies (Alexa fluor, 647 or 488; Invitrogen) were used, and DAPI was used to visualize cell nuclei.

### Statistical analysis

**Fig. 2:**
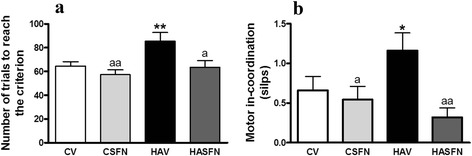
Treatment with sulforaphane restores learning ability in the Y maze and motor coordination in hyperammonemic rats. Control (C) and hyperammonemic (HA) rats treated with vehicle (V) or sulforaphane (SFN) were subjected to the conditional discrimination learning test in the Y maze (**a**) and the beam walking (**b**) tests. Values are the mean ± SEM of 11 rats per group in (**a**) and 22 rats per group in (**b**). Values significantly different from control rats are indicated by *asterisks*. Values significantly different from hyperammonemic rats are indicated by “*a*”. *p < 0.05; **p < 0.01; “*a*” *p* < 005; “*aa*” *p* < 0.01

**Fig. 3:**
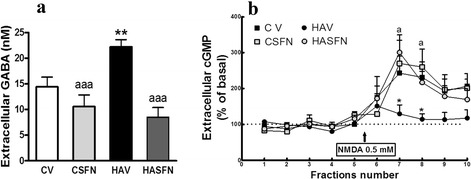
Sulforaphane normalizes extracellular GABA and the function of the glutamate-NO-cGMP pathway in the cerebellum of hyperammonemic rats. Control (C) and hyperammonemic (HA) rats treated with vehicle (V) or (SFN) were subjected to in vivo microdialysis in the cerebellum. **a** Extracellular GABA was measured by HPLC in the five initial samples of each rat. **b** After taking five samples to determine basal levels of cGMP, NMDA 0.5 mM was administered in the perfusion stream, and cGMP was determined in the following five samples to determine to function of the glutamate-nitric oxide-cGMP. Values are the mean ± SEM of six to eight rats per group. Values significantly different from control rats are indicated by *asterisks*. Values significantly different from hyperammonemic rats are indicated by “*a*”. **p < 0.01; “*a*” *p* < 0.05; “*aaa*” *p* < 0.001

**Fig. 4:**
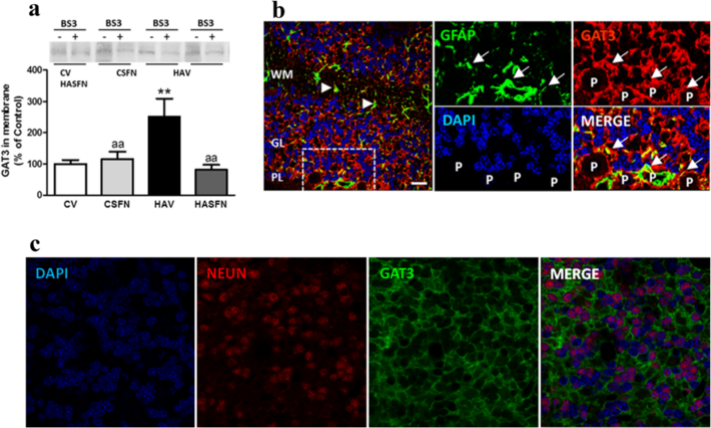
Hyperammonemia increases and treatment with sulforaphane normalizes membrane expression of GAT-3 in the cerebellum. **a** Membrane expression of GAT-3 was analyzed by Western blot after cross-linking with BS3. A typical image of the blots with and without BS3 is shown. Membrane expression was quantified as described in methods. Values are the mean ± SEM of six rats per group. Values significantly different from control rats are indicated by *asterisks*. Values significantly different from hyperammonemic rats are indicated by “*a*”. **p < 0.01; “*aa*” *p* < 0.01. **b** Double immunofluorescence staining of GFAP (*green*) and GAT-3 (*red*). Nuclei are stained with DAPI (*blue*). In the merged image, co-localization of GAT-3 and GFAP appears in *yellow*. It can be seen that GAT-3 is expressed in astrocytes surrounding Purkinje neurons (P). **c** Double immunofluorescence staining of Neun (*red*) and GAT-3 (*green*). Nuclei are stained with DAPI (*blue*). In the merged image, no co-localization of GAT-3 and Neun is observed

**Fig. 5:**
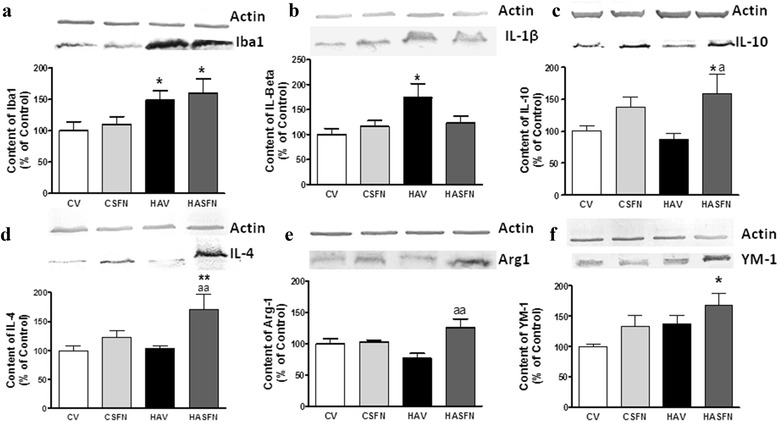
Hyperammonemia increases markers of M1 (pro-inflammatory) form of microglia and treatment with sulforaphane increases markers of M2 (anti-inflammatory) form of microglia in hyperammonemic rats. Cerebellar homogenates from control (C) and hyperammonemic (HA) rats treated with vehicle (V) or sulforaphane (SFN) were subjected to electrophoresis and Western blot using antibodies against **a** Iba1, marker of microglial activation; **b** the pro-inflammatory cytokine IL-1b, marker of M1 microglia; and against the markers of M2 microglia IL-10 (**c**), IL-4 (**d**), YM-1 (**e**), and Arginase 1 (**f**). Representative images of the blots are shown for each protein. Values are the mean ± SEM of six to nine rats per group. Values significantly different from control rats are indicated by *asterisks*. Values significantly different from hyperammonemic rats are indicated by “*a*”. *p < 0.05; **p < 0.01; “*a*” *p* < 0.05; “*aa*” *p* < 0.01

Results are expressed as mean ± SEM. In Figs. 2a, b, 3a, 4a, and 5a–f, data were analyzed by analysis of variance (one-way ANOVA) with Newman-Keuls multiple post hoc test. Two-way ANOVA was used in Fig. 3b, with fraction number and experimental group as variables. Significance levels were set at *p* < 0.05.

## Results

Blood ammonia levels were 18 ± 3 μM in control rats and were significantly (*p* < 0.001) increased in hyperammonemic rats to 54 ± 7 μM. Sulforaphane did not affect ammonia levels in control (18 ± 3 μM) or hyperammonemic (49 ± 6 μM) rats. These data show that the effects of sulforaphane are not due to reduction of hyperammonemia. The threefold increase in blood ammonia in hyperammonemic rats is similar to that reported in cirrhotic patients with minimal hepatic encephalopathy (2.64-fold) [33].

### Sulforaphane improves learning in the Y maze and motor in-coordination in hyperammonemic rats

Hyperammonemic rats show reduced ability to learn the Y maze task. They needed more trials (85 ± 7, *p* < 0.01) than control rats (65 ± 4) to learn the task. Treatment with SFN completely restored learning ability in hyperammonemic rats, which needed 64 ± 6 trials to learn the task. SFN did not affect learning ability in control rats, which needed 57 ± 4 trials to learn (Fig. 2a).

Motor coordination was analyzed with the beam walking. Hyperammonemic rats had more foot slips (1.16 ± 0.22, *p* < 0.01) than control rats (0.65 ± 0.17), indicating that they have motor in-coordination. Treatment with SFN restores motor coordination in hyperammonemic rats, reducing the number of foot slips to 0.32 ± 0.11. SFN did not affect motor coordination in control rats, which showed 0.54 ± 0.16 foot slips (Fig. 2b).

### Sulforaphane reduces extracellular GABA and restores the glutamate-NO-cGMP pathway in the cerebellum of hyperammonemic rats

We assessed the effects of treatment with SFN on extracellular GABA and on the function of the glutamate-NO-cGMP pathway by microdialysis in vivo in freely moving rats. As shown in Fig. 3a, extracellular GABA was increased in hyperammonemic rats (22 ± 2 nM; *p* < 0.01) compared to control rats (14 ± 2 nM). Treatment with SFN reduced extracellular GABA in hyperammonemic rats to 8.5 ± 1.9 nM (*p* < 0.01) and in control rats to 11 ± 2 nM.

Figure 3b shows the function of the glutamate-NO-cGMP pathway. The addition of NMDA through the microdialysis probe in fraction 6 activates the pathway in control rats increasing extracellular cGMP to 264 ± 51 and 231 ± 43 % of basal in fractions 7 and 8, respectively. The NMDA-induced increase in cGMP was strongly reduced (*p* < 0.05) in hyperammonemic rats to 129 ± 25 and 114 ± 15 % of basal in fractions 7 and 8, respectively, indicating reduced function of the glutamate-NO-cGMP pathway. SFN restores the pathway in hyperammonemic rats, and NMDA-induced increase of cGMP reached 300 ± 49 and 217 ± 31 % of basal, which was not different from control rats (Fig. 3b). SFN did not affect the pathway in control rats.

### Sulforaphane normalizes membrane expression of the GABA transporter GAT-3 and neuroinflammation in the cerebellum of hyperammonemic rats

A main modulator of extracellular GABA levels is the transporter GAT-3. As shown in Fig. 4a, the amount of GAT-3 in the membrane surface is strongly increased (*p* < 0.05) in hyperammonemic rats to 251 ± 58 % of control rats. Treatment with SFN normalized the surface expression of GAT-3 in hyperammonemic rats, reducing it to 82 ± 15 % of controls. SFN did not affect GAT-3 expression in control rats, which remained at 115 ± 24 % of controls (Fig. 4a).

**Fig. 6:**
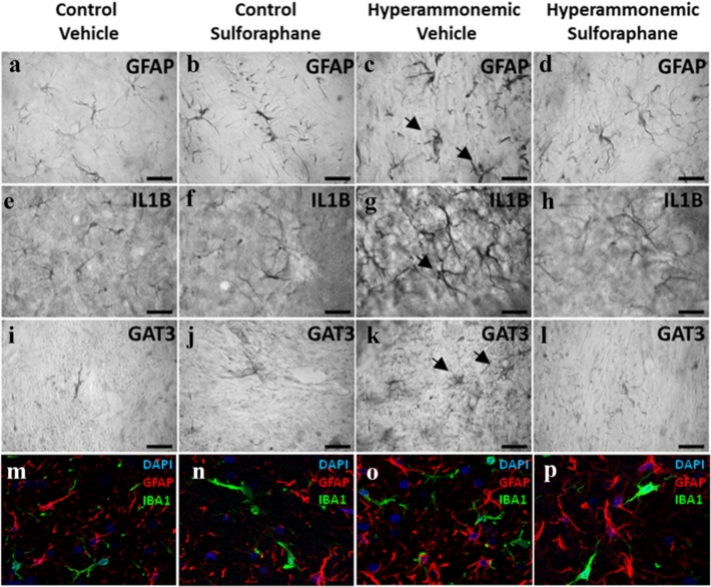
Hyperammonemia induces and treatment with sulforaphane reduces activation of astrocytes, which express IL-1b and GAT-3. Immunohistochemistry was performed as indicated in methods with DAB staining using antibodies against GFAP (**a**–**d**), IL-1b (**e**–**h**), or GAT-3 (**i**–**l**). Hyperammonemic rats show an altered morphology of astrocytes stained with GFAP (indicated by *arrows*), indicating activation (**c**). Treatment with SFN reduces astrocyte activation and normalizes the morphology (**d**). Activated astrocytes in hyperammonemic rats show increased labeling of IL-1b (**g**) and GAT-3 (**k**), which are normalized by treatment with SFN (**h**, **l**). **m**–**p** Double immunofluorescence staining of GFAP (*red*) and Iba-1 (*green*). Nuclei are stained with DAPI (*blue*). In the merged image, no co-localization of GFAP and Iba-1 is observed

Immunohistochemistry analysis showed that GAT-3 is expressed mainly in astrocytes surrounding Purkinje cells (Fig. 4b), and the increase in GAT-3 expression in hyperammonemia occurs mainly in activated astrocytes (Fig. 6k). No co-localization of GAT-3 and the neuronal marker Neun was observed when analyzed by double immunofluorescence labeling (Fig. 4c). This is in agreement with the literature showing that GAT-3 is expressed in astrocytes [34, 35].

We then assessed whether treatment with SFN reduces neuroinflammation and microglial activation in hyperammonemic rats. The marker of microglial activation Iba-1 was increased (*p* < 0.05) in hyperammonemic rats to 149 ± 15 % of controls. Treatment with SFN did not affect Iba-1 content in control or hyperammonemic rats (Fig. 5a).

The pro-inflammatory cytokine IL-1β is increased in the cerebellum of hyperammonemic rats to 173 ± 28 % (*p* < 0.05) of the control rats. Treatment with SFN normalizes IL-1β in hyperammonemic rats to 123 ± 14 % of controls and did not affect IL-1β in control rats, remaining at 116 ± 12 % of control (Fig. 5b).

A main effect of SFN in hyperammonemic rats was the induction of anti-inflammatory cytokines IL-10 and IL-4. IL-10 levels were not significantly altered in the cerebellum of hyperammonemic rats (88 ± 9 % of controls). However, treatment with SFN increased (*p* < 0.05) IL-10 in hyperammonemic rats to 159 ± 29 % of control rats (Fig. 5c). SFN also tended to increase IL-10 in control rats (137 ± 16 % of controls), but the effect was not statistically significant.

A similar effect was observed for IL-4. IL-4 levels were not significantly altered in the cerebellum of hyperammonemic rats (104 ± 4 % of controls). Treatment with SFN strongly increased (*p* < 0.01) IL-4 in hyperammonemic rats to 171 ± 26 % of control rats (Fig. 5d). SFN did not affect IL-4 in control rats (112 ± 12 % of controls).

These data show that SFN reduces pro-inflammatory and increases anti-inflammatory cytokines. To assess whether this could be due to promotion of M2 microglia polarization, we measured the amount of two main markers of M2 microglia: arginase 1 and YM-1. Their amount in the cerebellum was not altered in hyperammonemic rats or in control rats treated with SFN. However, in hyperammonemic rats, treatment with SFN induces a significant (*p* < 0.05) increase of arginase 1 (125 ± 14 % of controls, Fig. 5f) and especially of YM-1, which increased to 168 ± 21 % of controls (Fig. 5e), indicating M2 polarization of microglia.

The total amount of GFAP, as analyzed by Western blot, was not affected by hyperammonemia or SFN treatment. However, astrocytes in hyperammonemic rats show altered morphology (Fig. 6c) compared to controls (Fig. 6a) when stained with GFAP, indicating astrogliosis, an activation of astrocytes which is not reflected in the total amount of GFAP analyzed by Western blot. In hyperammonemic rats treated with SFN, the morphology of astrocytes is similar to controls, indicating that SFN reduces activation of astrocytes.

**Fig. 7:**
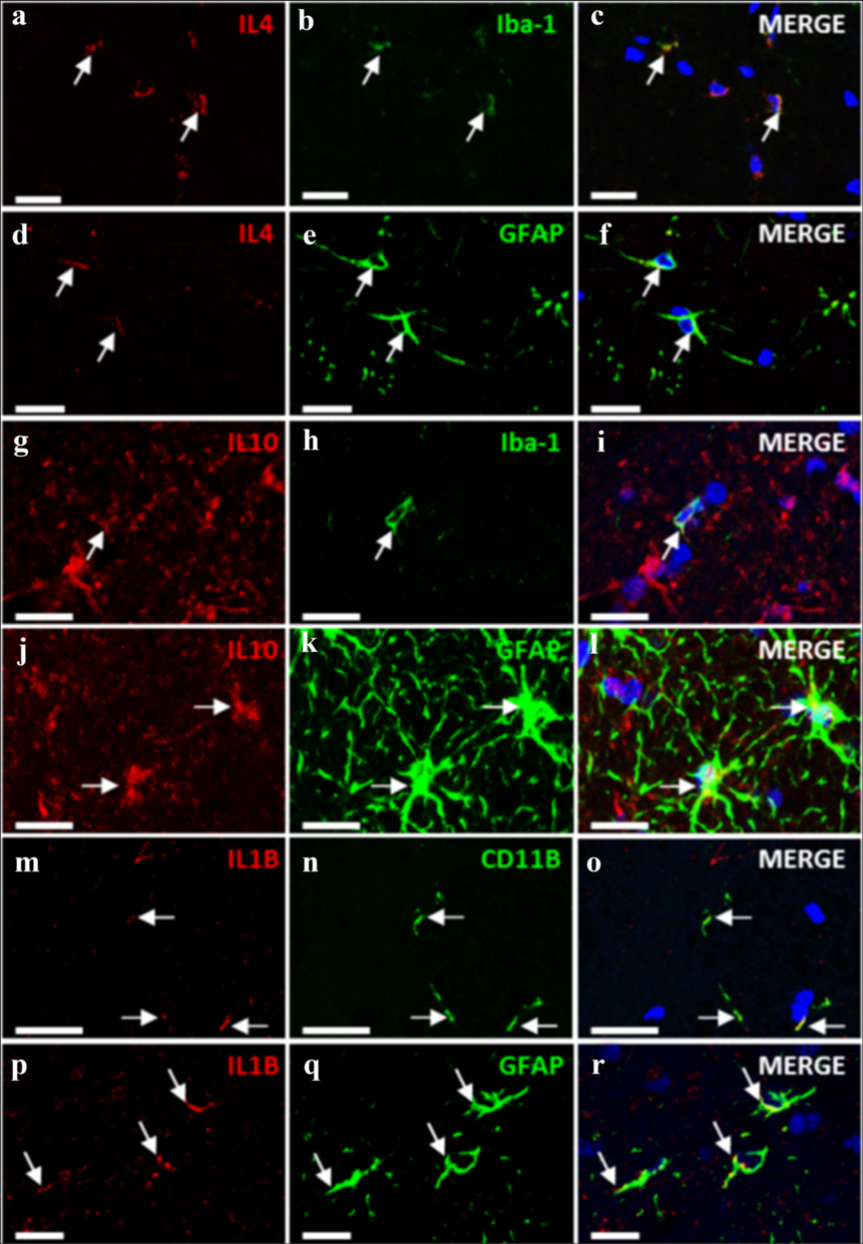
IL-4, IL-10, and IL-1b are present both in microglia and astrocytes. The images show immunostaining by double fluorescence of astrocytes (GFAP) or microglia (Iba-1 or CD11b) and IL-4 (**a**–**f**); IL-10 (**g**–**l**), or IL-1b (**m**–**r**). *Arrows* indicate co-localization of the labeling of the IL with the corresponding cell type marker

To assess in which cell types are IL-1b, IL-4, and IL-10 expressed, we performed co-localization studies by immunohistochemistry using antibodies against these ILs and against markers of astrocytes (GFAP) or microglia (Iba-1 and CD11B). The results are shown in Fig. 7. IL-4 co-localizes both with Iba-1 (Fig. 7a–c) and with GFAP (Fig. 7d–f) indicating that it is expressed both in astrocytes and in microglia. The same occurs with IL-10 and IL-1b, which co-localize both with microglia (Fig. 7g–i and m–o, respectively) and astrocytes (Fig. 7j–l and p–r, respectively). As shown in Fig. 6g, in hyperammonemic rats, IL-1b is increased in activated astrocytes and treatment with SFN reduces it.

## Discussion

The two main contributions of this article are that it shows, for the first time, (a) a definite link between neuroinflammation, altered GABAergic neurotransmission, and neurological alterations and (b) the beneficial effects of sulforaphane to improve hyperammonemic encephalopathy. These contributions are discussed in detail below.

### Mechanisms by which hyperammonemia-induced neuroinflammation leads to cognitive and motor impairment

**Fig. 8:**
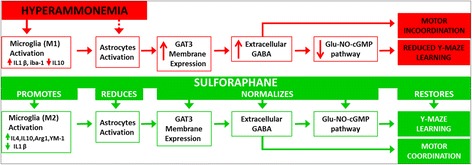
Proposed sequences of events by which hyperammonemia-induced neuroinflammation leads to cognitive and motor impairment (in *red*) and treatment with sulforaphane restore them (in *green*). See details in “Discussion” section

The results reported provide relevant new data on the mechanisms by which hyperammonemia-induced neuroinflammation leads to cognitive impairment and motor in-coordination and on how treatment with SFN reverse them. These data allow proposing the sequence of events shown in Fig. 8, which can be summarized as follows. Hyperammonemia induces activation of microglia and of astrocytes which results in increased levels of Iba1, a marker of microglial activation, and of the pro-inflammatory IL-1b. This neuroinflammation leads to enhanced membrane expression of GAT-3, resulting in enhanced release of GABA and increased extracellular GABA concentration in the cerebellum. The increase in GABA leads to motor in-coordination and to reduced function of the glutamate-NO-cGMP pathway which, in turn, impairs the ability to learn the Y maze task. Impairment of the glutamate-NO-cGMP pathway and reduction of extracellular cGMP by activation of GABA_A_ receptors in the cerebellum have been already reported by Fedele et al. [43] and Cauli et al. [32].

A main contribution of this part of the study is that it provides a mechanistic link between neuroinflammation and enhanced GABAergic tone in hyperammonemia: the increased membrane expression of GAT-3.

Recent studies have clearly shown that hyperammonemia induces neuroinflammation, with microglial activation, that contributes to cognitive and motor alterations in rat models of hyperammonemia that can be reversed with anti-inflammatories [8]. Microglial activation has also been shown in rat models of HE including portacaval shunt or bile duct ligation (BDL) [19, 20, 44, 45]. Wright et al. [46] reported that bile duct ligation triggers early alternative, but not classical, microglial activation. However, several other groups report classical microglial activation in BDL rats [8, 44, 45]. Microglial activation is therefore a general effect in rat models of chronic hyperammonemia and HE.

On the other hand, hyperammonemia also leads to increased levels of extracellular GABA and enhanced GABAergic tone in the cerebellum which impairs the function of the glutamate-NO-cGMP pathway, resulting in reduced ability to learn the Y maze task [32]. Increased extracellular GABA in the cerebellum also contributes to motor in-coordination. Reducing extracellular GABA and GABAergic tone by modulating GABA_A_ receptors with the antagonist bicuculline or with the neurosteroid pregnenolone sulfate restores learning ability and motor coordination [32, 33].

However, it was not known how these two processes induced by hyperammonemia (neuroinflammation and enhanced GABAergic tone) are connected. We show here that neuroinflammation leads to enhanced membrane expression of GAT-3, mainly in activated astrocytes surrounding Purkinje cells, which would be responsible for the increased levels of extracellular GABA and of enhanced GABAergic tone which leads both to impaired learning in the Y maze and to motor in-coordination in the beam walking.

An increase in the total amount of GAT-3 induced by pro-inflammatory cytokines such as IL-1b has been reported in the parietal cortex, hippocampus, and amygdala of rats injected with kainate [47]. Under normal conditions, GAT-3 transports GABA from the extracellular space into astrocytes. However, this transport may be reversed under pathological conditions. Wu et al. [48] showed that a mouse model of Alzheimer’s disease (5xFAD) show activated astrocytes in the hippocampus which release GABA through GAT-3 transporters. Blocking GAT-3 transporters reduced GABA concentration and tonic GABA currents while blocking GAT-1 transporters enhanced GABA currents. This supports that, under these pathological conditions, GAT-3 is releasing while GAT-1 is uptaking GABA [48]. A similar GAT-3-mediated release of GABA would occur in activated astrocytes in the cerebellum of hyperammonemic rats.

These reports, together with the results reported here, suggest that enhanced GABAergic tone in certain brain areas may be a general consequence of neuroinflammation which would contribute to the neurological alterations associated to different neurological and neurodegenerative diseases. Moreover, we show that neuroinflammation enhances the membrane expression of GAT-3. This is very relevant because only GAT-3 in the membrane can release GABA to the extracellular space.

This study provides therefore a mechanistic link (modulation of membrane expression of GAT-3) between neuroinflammation and increased GABAergic tone and neurological alterations in chronic hyperammonemia and HE. Further detailed analysis of the mechanisms by which neuroinflammation enhances transport of GAT-3 to the membrane could provide new therapeutic targets for the cognitive and motor alterations not only in hyperammonemia and HE but also in other pathologies associated to neuroinflammation, including some neurodegenerative diseases.

### Treatment with sulforaphane restores learning ability and motor coordination. Underlying mechanisms

Another main contribution of this work is the identification of a new therapeutic tool, sulforaphane, to improve cognitive and motor function in hyperammonemia and hepatic encephalopathy. Moreover, we also identify mechanisms underlying the beneficial effects of sulforaphane.

Figure 8 also summarizes the mechanism and steps by which treatment with SFN restores the ability to learn the Y maze task and motor coordination. The main effect of SFN is to promote differentiation of microglia from the pro-inflammatory M1 form to the anti-inflammatory M2 form (see below). This effect is not observed in controls. This may be attributed to the fact that in control rats, microglia is not activated, remaining in resting state, not reaching M1 phenotype. It is not possible therefore to promote its differentiation from M1 to M2.

Differentiation of microglia from the M1 to the M2 form in hyperammonemic rats is associated with a reduction of the pro-inflammatory IL-1b and of astrocytes activation. Increased expression of GFAP is a hallmark of activated astrocytes [49, 50]. The intensity of GFAP immunostaining is increased in astrocytes of hyperammonemic rats and is normalized by treatment with sulforaphane, indicating reversal of astrocytes activation of hyperammonemic rats by sulforaphane.

As a result of reduced neuroinflammation, pro-inflammatory cytokines, and astrocytes activation, the levels of GAT-3 in the membrane return to normal levels. As a consequence, extracellular GABA levels also return to normal levels, allowing normalization of the glutamate-NO-cGMP pathway and restoration of learning ability. The normalization of extracellular GABA levels also leads to improvement of motor coordination.

The fact that SFN, by promoting differentiation of microglia from the pro-inflammatory M1 form to the anti-inflammatory M2 form, normalizes all the subsequent steps of the process summarized in Fig. 8 further supports the validity of the sequence of events proposed for hyperammonemia and HE.

It should be noted that in the present work, there is a gap between behavioral analysis (performed at weeks 4–6 of hyperammonemia), microdialysis studies (performed at weeks 6–7), and the sacrifice (performed at week 8 (Fig. 1)). The severity of encephalopathy may differ from the time of behavioral and microdialysis analysis and the time of sacrifice. However, we believe that this gap is small and would not affect the main conclusions of the study, summarized in Fig. 8.

Microglial activation is the principal component of neuroinflammation in the brain, which execute both detrimental and beneficial effects on the neurons in many pathological situations. Microglia may present diverse functional phenotypes that range from pro-inflammatory M1 phenotype, the first line of defense, to immunosuppressive M2 phenotype, which includes alternative activation and acquired deactivation [51]. Markers of M1 phenotype are IL-1b or nitric oxide and markers of M2 are Il-4, IL-10, arginase 1 (Arg 1), and YM-1 [51]. It has been proposed that manipulation of microglia phenotypes from pro-inflammatory, cytotoxic M1 to anti-inflammatory, neuroprotective M2 may be a therapeutic approach in some neurodegenerative diseases associated with neuroinflammation such as Alzheimer’s or Parkinson’s diseases and amyotrophic lateral sclerosis [51].

The results reported here show that treatment with sulforaphane successfully promotes this polarization of microglia to neuroprotective M2 phenotype in cerebellum of hyperammonemic rats, increasing M2 markers (IL-4, IL-10, Arg 1, and YM-1) and reducing M1 markers (IL-1b).

This is associated with improvement of cognitive function and motor coordination which would be mediated by deactivation of astrocytes. In pathological conditions, there is a cross-talk between activation of microglia and astrocytes. Microglia is activated earlier than astrocytes and promotes activation of astrocytes, which is mediated mainly by IL-1b released from microglia [52]. As mentioned above, activated astrocytes show increased amount and membrane expression of GAT-3, resulting in increased release and extracellular levels of GABA which in turn induces motor in-coordination and cognitive impairment.

As summarized in Fig. 8, we show that treatment with sulforaphane promotes the M2 phenotype, reducing microglial activation and normalizing IL-1b levels. This is associated with deactivation of astrocytes and normalization of membrane expression of GAT-3 and of extracellular GABA levels. This normalizes GABAergic neurotransmission and restores motor coordination and learning ability.

The above beneficial effects of sulforaphane would be due to a direct effect on the brain. In the rat model used, chronic hyperammonemia is obtained by feeding rats an ammonium-containing diet. In this model, there is no liver damage [53]. The effects of sulforaphane cannot be due therefore to improvement of liver failure. Sulforaphane is a natural compound present in cruciferous vegetables such as broccoli. In addition to pharmacological treatments with compounds that promote differentiation of M1 microglia to M2, maybe including these vegetables in the diet of patients may help to improve their cognitive and functional status.

## Conclusions

In summary, this work shows for the first time that hyperammonemia-induced activation of microglia, neuroinflammation, and increased extracellular GABA are associated with increased levels (mainly in activated astrocytes) and membrane expression of GAT-3, that releases GABA from cells to the extracellular fluid. Increased GABAergic tone has been shown to mediate impairment of motor coordination and of learning of the Y maze task in hyperammonemia and HE. Treatment with sulforaphane restores this learning ability and motor coordination by promoting the differentiation of microglia from the M1 to the M2 phenotype, reducing the levels of IL-1b and de-activating astrocytes. This is associated with reduced membrane expression of GAT-3 and extracellular GABA. The process depicted in Fig. 8 linking neuroinflammation with enhanced GABAergic tone and impaired learning ability and motor coordination would occur not only in hyperammonemia and HE but also in other pathological situations leading to neuroinflammation, including some neurodegenerative diseases. Promoting differentiation of microglia from M1 to M2 phenotypes, with sulforaphane or other compounds, could be a new therapeutic approach to improve cognitive and motor function in these situations, including hyperammonemia and HE.
